# Impact and experiences of delayed discharge: A mixed‐studies systematic review

**DOI:** 10.1111/hex.12619

**Published:** 2017-09-12

**Authors:** Antonio Rojas‐García, Simon Turner, Elena Pizzo, Emma Hudson, James Thomas, Rosalind Raine

**Affiliations:** ^1^ NIHR CLAHRC North Thames Department of Applied Health Research University College London London UK; ^2^ Institute of Education EPPI‐Centre University College London London UK

**Keywords:** cost, delayed discharge, impact, OECD, outcome, qualitative, systematic review, timely discharge

## Abstract

**Background:**

The impact of delayed discharge on patients, health‐care staff and hospital costs has been incompletely characterized.

**Aim:**

To systematically review experiences of delay from the perspectives of patients, health professionals and hospitals, and its impact on patients’ outcomes and costs.

**Methods:**

Four of the main biomedical databases were searched for the period 2000‐2016 (February). Quantitative, qualitative and health economic studies conducted in OECD countries were included.

**Results:**

Thirty‐seven papers reporting data on 35 studies were identified: 10 quantitative, 8 qualitative and 19 exploring costs. Seven of ten quantitative studies were at moderate/low methodological quality; 6 qualitative studies were deemed reliable; and the 19 studies on costs were of moderate quality. Delayed discharge was associated with mortality, infections, depression, reductions in patients’ mobility and their daily activities. The qualitative studies highlighted the pressure to reduce discharge delays on staff stress and interprofessional relationships, with implications for patient care and well‐being. Extra bed‐days could account for up to 30.7% of total costs and cause cancellations of elective operations, treatment delay and repercussions for subsequent services, especially for elderly patients.

**Conclusions:**

The poor quality of the majority of the research means that implications for practice should be cautiously made. However, the results suggest that the adverse effects of delayed discharge are both direct (through increased opportunities for patients to acquire avoidable ill health) and indirect, secondary to the pressures placed on staff. These findings provide impetus to take a more holistic perspective to addressing delayed discharge.

## 1 BACKGROUND

Delayed discharge is an important problem for health‐care providers internationally.^1^, ^2^, ^3^ It is defined as the period of continued hospital stay after a patient is deemed medically fit to leave hospital but is unable to do so for non‐medical reasons.^4^ Costs to the National Health Service (NHS) in England associated with delayed discharge are approximately £100 m per year^5^ and resulted in 1.2 million bed‐days lost in 2013‐14.^3^ A Canadian study found that between 8 and 10% of beds in acute hospitals were occupied inappropriately by delayed patients.^2^

Delayed discharge is recognized to be a system‐level problem requiring effective team working within hospitals and coordination between health and social care.^6^, ^7^, ^8^ However, an in‐depth understanding of the impact of delayed discharge on patients and the health‐care staff caring for them needs to be established so that managers and policymakers can make informed decisions about addressing the consequences of delays. The costs of delayed discharge to hospitals, the health and social care system, and patients and carers also need to be understood. This systematic review assesses the impact and experiences of delayed discharge at multiple levels, from the perspective of patients, health professionals and hospitals; and associated costs of delay.

This review systematically examines quantitative and qualitative studies to (i) quantify the impact of delayed discharge on health outcomes, (ii) qualitatively assess impacts on patients, health professionals and provider organizations, and (iii) evaluate the potential costs associated with delay. Studies conducted in OECD countries^9^ were included to examine delayed discharge across health systems in countries with comparable economic development.

## 2 METHODS

This review is reported using the Preferred Reporting Items for Systematic Reviews and Meta‐Analyses (PRISMA) guidelines^10^ (Appendix S4), and the protocol is published in PROSPERO (CRD42016035256).^11^

### 2.1 Information sources and search strategies

Studies were identified using 6 biomedical databases (as below) which were searched in February 2016 for the period 2000‐2016. The searches were limited to publications dated from 2000 onwards to ensure the studies are relevant to contemporary health systems. Specific search strategies were designed for Medline, Embase, CINAHL, PsycINFO, HMIC (Health Management Information Consortium) and NHS EED. The initial search was designed for Medline (via Ovid), which combined MeSH terms and keywords, and later adapted to other databases (Appendix S1), including search terms such as “delayed discharge,” “timely discharge,” “unnecessary days” and “inappropriate stays.” This search was complemented with grey literature sources and consulting other systematic reviews and original papers. A bibliographic database was created to manage the references using EPPI‐Reviewer 4.^12^

### 2.2 Study selection

Included studies addressed the impact and experiences of delayed discharge. Studies were included where they met one or more of the following inclusion criteria: (i) quantitative data on the impact of delayed discharge on health outcomes (eg quality of care, patient satisfaction, number of infections, mental health, mortality, morbidity, readmissions and functioning), (ii) qualitative data on experiences of delay from perspectives of patients (eg perceived impact on physical health or patient experience), health professionals (eg affect on staff role and working relationships) and hospitals (impacts at the organizational level, eg costs of managing delays and affect on culture), and (iii) information on costs of delay due to unnecessary bed‐days. Furthermore, only studies written in English, published since 2000 and conducted in the OECD were included.

The following exclusion criteria were also applied to the articles identified through the database search: research focusing on mental health, maternal and child and adolescent health, and palliative care was excluded; delays may occur in those settings for different reasons, for example relapse of mental health disorders,^13^, ^14^ and consequently, delays due to non‐medical reasons are difficult to determine.^14^, ^15^, ^16^, ^17^ Abstracts, editorials, commentaries and book reviews were excluded because the review focused on primary research.

### 2.3 Assessment of eligibility

The title/abstract of references were screened for eligibility by 2 reviewers, and then, the full text of those references which fulfilled the inclusion criteria was assessed. Discussion with a third reviewer was used to resolve disagreements.

### 2.4 Quality assessment

We determined the quality of the quantitative studies using a standardized tool for assessing the methodological quality of quantitative/observational studies.^18^ The focus of some questions was adapted to ensure their relevance to the topic, covering the following fields: control group, confounders, sample, measures, reliability and relevance in a health service context (Appendix S2).

The qualitative studies were quality‐assessed using 6 criteria of the “weight of evidence” (with respect to reliability and usefulness) developed by the EPPI‐Centre^19^, ^20^ (Appendix S2). A score (low, medium, high) was then allocated to each study. Reliability was based on assessment of rigour in study sampling, data collection, analysis and findings. Usefulness was based on assessment of the breadth and depth of findings and the extent to which the perspectives of health‐care professionals and patients/carers’ perspectives were prominent in the studies.

We used the checklist for the critical assessment of economic evaluation^21^ and the NICE guide on methods of technology appraisal^22^ to select and appraise the quality of health economic studies.

### 2.5 Data extraction and synthesis of the results

The following characteristics were summarized for each study: design, setting, year of publication, country, target population, sociodemographic characteristics, disease(s) and reason(s) for delayed discharge (Appendix S3). For the quantitative and health economic studies, results were classified into categories depending on the nature of the outcome. Experiences of delay reported in the qualitative studies were divided into 3 categories: (i) perceptions of patients, (ii) perceptions of health professionals, and (iii) experiences of delay for hospitals.

## 3 RESULTS

### 3.1 Identification of the studies

**Figure 1:**
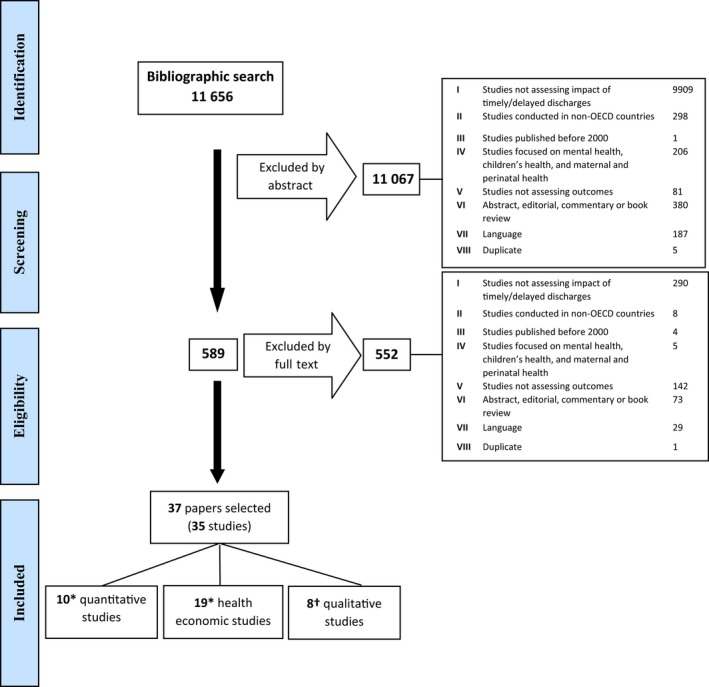
PRISMA flow chart of the selection process for the delayed discharge review. *Two studies provided data on costs and quantitative variables.^30^, ^32^^**†**^Three papers reported data from one study^35^, ^36^, ^37^

The search retrieved 11 656 references. After conducting the title and abstract screening, 589 references were included for full‐text assessment. A total of 552 studies had to be excluded mostly because they did not consider experiences, impact or outcomes of delay (Figure 1), leaving 37 papers included in the review, reporting data on 35 studies.

### 3.2 Characteristics of the studies

The study characteristics are summarized in Table 1. There were 10 quantitative, 8 qualitative and 19 health economic studies. More than half the studies were undertaken in the UK (14) and the United States (8). Half the studies analysed data across different service areas, and others focused on 1 type of service, for example trauma (5), acute (4) and intensive care (4). Thirteen studies examined elderly patients only.

**Table 1:** Characteristics of studies

	N = 35	%
Country		
The UK	12	34.29
The USA	6	17.14
Canada	2	5.71
The Netherlands	2	5.71
Spain	2	5.71
France	2	5.71
Switzerland	2	5.71
Rest of OECD Countries	7	20.00
Type of service(s) / unit(s)		
General	18	51.43
Trauma	5	14.29
Acute care	4	11.43
Orthopaedics	3	8.57
Others (ie Rehabilitation)	3	8.57
Not reported	18	51.43
Target population		
Only 60 y or older	13	37.14
Adult population	18	51.43
Health professionals^a^	1	2.86
Not reported	3	8.57

a Only applicable for qualitative studies.

### 3.3 Quality assessment

Three of the quantitative studies were deemed to have high methodological quality,^23^, ^24^, ^25^ 4 moderate quality^26^, ^27^, ^28^, ^29^ and 3 low quality^30^, ^31^, ^32^ (Table 2). Two of the eight qualitative studies were removed due to low reliability and usefulness,^33^, ^34^ as determined using the 6 criteria for assessing study quality (Table 3).

**Table 2:** Summary of the quantitative studies

Authors	Country	Study design	Type of unit(s)/service(s)	Target population	Summary of the results	Methodological quality
Carter, (2002)^30^	The UK	Cross‐sectional study	Acute care	Patients with delayed discharge	Twenty‐six (52%) patients showed cognitive impairment (SOMC <18/28). Thirty‐nine (78%) patients had a Barthel Index score of less than 15/20 and 24 (48%) of less than 10/20. There was no correlation between the period of discharge delay and cognitive impairment, motricity or activities of daily living. Only 9 patients had suitable accommodation at discharge	Low
Challis, (2014)^23^	The UK	Cohort (retrospective) study	Not reported	Patients were identified from local arrangements according to the Community Care (Delayed Discharges, etc.) Act 2003, as part of the “SitReps” reporting system	Using bivariate analysis, cognitive impairment and dependency were significantly correlated with delays in discharge. The multivariate analysis showed that dependence and cognitive impairment had a different impact on delay and LOS	High
Costa, (2012)^28^	Canada	Retrospective cohort study	Acute care	Admissions to acute hospital identified as Alternate Level of Care (ALC)	Morbid obesity, psychiatric diagnosis, abusive behaviours, and stroke were characteristics significantly associated with greater ALC lengths of stay	Moderate
Hwabejire, (2013)^32^	The USA	Cross‐sectional study	Trauma unit	Patients (>18 y) admitted to the Massachusetts General Hospital's trauma unit	There were no statistically significant differences regarding Injury Severity Scores, Revised Trauma Scores and in‐hospital complication rates	Low
Ingold, (2000)^26^	Switzerland	Prospective cohort study	Internal medicine	Patients aged >75	Univariate analysis found that patients with inappropriate stays were more impaired in activities of daily living (ADL), and more frequently had depressed symptoms. Multivariate analysis showed independent associations for subjects living alone, those with depression, with basic ADL dependencies, and Instrumental ADL dependencies	Moderate
Jasinarachchi, (2009)^27^	The UK	Prospective observational study	District general hospital	Elderly patients (>65 y) who were delayed	Five of the 18 inpatient deaths happened during the delay. Seven patients suffered medical complications during the delay. The number of inappropriate extra bed in this study was 682 d (mean = 4.8)	Moderate
Moeller, (2006)^25^	Canada	Cross‐sectional study	Not reported	Patients were included in the study if they met the following criteria: primary diagnosis of community‐acquired pneumonia and admission through the emergency department	No differences were found between patients discharged when stable and patients with longer length of stay in relation to demographics, pneumonia severity score, functional or cognitive status at discharge using the Barthel Index and MMSE. There was a significant difference in mobility (HABAM) at the time of clinical stability, which was associated with patients’ readiness for discharge as assessed by the physician and family	High
Rosman, (2015)^29^	Israel	Retrospective cohort study	Internal Medicine Departments	Those patients diagnosed as suffering from stroke and those recovering from severe or acute illness	In‐hospital mortality or hospital‐acquired infection happened in 32 patients (31%). The first 3 inappropriate days was the most harmful time where 63.7% of patients experienced a medical condition and 44% of the total number of complications took place during this length of time. There was a statistically significant association between the presence of any complication during the inappropriate stay and greater risk of mortality during the first year after discharge	Moderate
Umarji, (2006)^31^	England	Cross‐sectional study	A regional trauma centre	All consecutive patients older than 60 y with proximal femoral fracture	Nosocomial infection occurred in 99 patients (58%) when inappropriate stay lasted more than 8 d (after surgery). A total of 145 patients (85%) obtained their maximum mobility score by the 8th day and 162 patients (95%) by the 10th post‐operative day. There was no benefit in patients staying in hospital more than 8 d and most acquired nosocomial infection following this period	Low
Young, (2010)^24^	The UK	Results from RCT	Five urban and rural centres with rehabilitation	Patients were eligible for the study if, after an acute admission to a general hospital, they were medically stable and considered by their responsible clinician to need post‐acute rehabilitation care	Differences were found between the “early transfer” (87 patients), late transfer (78 patients) and control (“no transfer”) (121 patients) groups for changes in activities of daily living (Nottingham Extended Activities of Daily Living Scale) from baseline to 6 mo	High

**Table 3:** Findings from qualitative studies and assessment of the reliability and usefulness of findings

Study	Country	Themes				Weight of evidence	
		How experiences of delay affect physical health	Impact on patient experience	Experiences of staff	Experiences of hospitals	Reliability	Usefulness
DH (2004)^33^, ^a^	The UK					Low	Low
Connolly (2009)^41^	The UK	Delayed patients transferred across wards to accommodate new patients; physical or emotional effect can set back their overall discharge date from the hospital	Patients “systematized” or “dehumanized” by discharge planning arrangements, according to staff	Some staff preoccupied with discharging patients, rather than providing care to those at hand Potential differences in staff groups’ attitudes towards discharging patients (external pressure on manag ers and senior doctors to discharge) Effective working relationships, including information sharing, undermined by delays Where the environment is pressured due to need to reduce waiting lists, nursing staff may “forget to do things.”	For nursing staff, costs include producing reports and making phone calls to arrange discharge	Medium	High
Cornes (2008)^35^ Godfrey (2008)^36^ Godfrey (2009)^37^	The UK	Discharge delays may increase the risk of acquiring infections and bed sores	Wards can be noisy even at night and lack personal privacy Emotional effects of delay included tedium or boredom, depression, and loss of independence Pressure placed on patients and their families to arrange care outside the hospital	Delayed discharge has an impact on interprofessional relationships. For instance, 1 hospital social worker described the “extra pressure” and “flak” they received from other staff in relation to delays	Interventions create administration costs; 1 social services manager referred to the emergence of “a whole industry around delayed discharge.”	High	High
Ekdahl (2012)^40^	Sweden	Bed shortages create pressure to discharge patients, which has potential consequences for their physical health, for example patients reported not recovering sufficiently before discharge		The need to reduce waiting lists for treatment within hospital, created pressure for some patients to be discharged home, which in turn created frustration and guilt among staff		Medium	Medium
Fuji (2013)^39^	The USA	Where discharge is rushed, patient needs may not be addressed effectively	Rushed discharge may cause patients worry and dissatisfaction with services, particularly where they feel unable to ask questions or clarify information			Medium	Low
Gansel (2010)^34^, ^a^	France					Low	Low
Kydd (2008)^43^	The UK		Boredom while delayed; anxiety felt about further transfers within or outside hospital	Some staff preoccupied with discharging patients, rather than providing care to those at hand	The issue of delayed discharge may contribute to a “poor mood on the ward” which can have a knock‐on effect on patients Where the environment is pressured, nursing staff may lack the emotional capacity to provide a “cheerful environment.”	Low	Medium
Swinkels (2009)^38^	The UK	Patients express concern about deterioration in their general health while in hospital (eg due to limited opportunities for movement)	Wards can limit patient independence, contributing to boredom, frustration, and low mood Consequence of physical and emotional impact of delays is disengagement from discharge planning processes, with some patients too unwell or withdrawn to engage Staff being busy and difficult to engage has negative effect on patients			High	High

a Removed due to low methodological quality.

In this review, health economic studies refer to those studies reporting on cost of delay. These studies were quantitative and looked at cost implications of delayed discharge. There were 2 studies^30^, ^32^ reporting data on costs and health outcomes, both deemed with low methodological quality.

### 3.4 Summary of the quantitative studies

The characteristics and methodological quality of the ten quantitative studies are summarized in Table 2. Seven cohort studies, either prospective (3) or retrospective (4), and 3 cross‐sectional studies were identified. Eight studies used checklists (eg the Appropriateness Evaluation Protocol, AEP) or health professionals’ criteria to identify patients who were delayed for non‐medical reasons.^23^, ^25^, ^26^, ^28^, ^29^, ^30^, ^31^

#### 3.4.1 Impact of delayed discharge on health outcomes

Ten studies explored the impact of delayed discharge on health outcomes. These studies typically carried out assessments at 2 time points (at baseline and at discharge or during the delay period), and some compared the results to non‐delayed patients.^24^, ^25^, ^26^, ^32^ Two studies explored the factors associated with delayed discharges and inappropriate stays in hospital.^23^, ^28^

The potential impact of delayed discharge on mortality was examined in 2 studies with moderate methodological quality. One study found that 5 of the 58 patients suffering delayed discharge (8.6%) died in hospital after they were medically fit for discharge.^27^ The other demonstrated a significant association between increased risk of mortality and inappropriate stay during the first year after discharge.^29^

A prospective study conducted in a district general hospital in the UK which focused on patients over 65 years old, with moderate methodological quality, found that 7 of 58 cases of delayed discharge (12.1%) developed at least 1 medical complication prolonging their hospitalization.^27^ Conditions included “urinary tract infection, recurrent dizziness, leg swelling, poor oral intake, lower respiratory tract infection, bronchopneumonia and Clostridium difficile diarrhoea.” A retrospective cohort study conducted in Israel^29^ with moderate methodological quality showed that among patients who had been medically fit for discharge, 9 (8.7%) suffered from pneumonia; 14 (13%) suffered from urinary tract infection; 9 had sepsis (8.7%); and 1 (0.96%) patient acquired Clostridium difficile during the inappropriate stay. Another UK study with low methodological quality assessed consecutive patients who sustained proximal femoral fracture over 60 years of age and found that nosocomial infection happened in 58% of patients (99 patients) when inappropriate stay lasted longer than 8 days.^31^

Two studies, with high^24^ and moderate^26^ methodological quality, respectively, evaluated depression and anxiety, one of which found statistically significant differences in levels of depressive symptoms in patients with delays in discharge.^26^

Five studies examined impact on daily living activities/mobility, 3 of which had high methodological quality. A UK study^24^ found that patients with delayed transfer between hospitals presented worse scores on activities of daily living. In Canada, a cross‐sectional study found that there was a significant difference in the Hierarchical Assessment of Balance and Mobility (HABAM) score when clinical stability was achieved the first year after the inappropriate hospital stay.^25^ A prospective cohort study that took place in Switzerland found that delayed discharge patients became more impaired in daily living activities, either basic or instrumental, during the prolonged stay.^26^ Two UK studies with moderate^27^ and high^23^ methodological quality showed that delayed discharge had a negative impact on mobility and daily living activities.

### 3.5 Summary of the qualitative studies

#### 3.5.1 Synthesis of results from qualitative studies

Features of the 6 qualitative studies, including quality assessment, are summarized in Table 3. Four studies were undertaken in the UK, 1 in the United States and 1 in Sweden. Five studies were conducted in hospital settings; only 1 examined the coordination of care across multiple settings. The results from the 6 qualitative studies are divided into 4 themes concerning experiences of delay from the perspectives of: (i) patient experience, (ii) patients’ physical health, (iii) staff/health professionals, and (iv) hospitals.

#### 3.5.2 Impact on patient experience

The data on patient experience was derived from interviews with patients and health professionals. Delays in discharge affected patients’ emotional state. Hospitals were considered poor environments for a protracted stay because wards could be noisy even at night, they lack personal privacy^35^, ^36^, ^37^ and they limit patients’ autonomy.^38^ A knock‐on effect of delayed discharge was pressure on hospitals to expedite other patients’ discharge. Where discharge was rushed to free up beds, this could cause patients to worry and become dissatisfied with services, particularly when they felt unable to ask questions.^39^ It also sometimes led to disengagement from discharge planning.^38^

Patients expressed anxiety and other negative feelings about delays. Emotional outcomes of delay included tedium or boredom, depression and loss of independence.^35^, ^36^, ^37^ One elderly patient awaiting assessment on a stroke rehabilitation ward communicated a sense of desperation and reported being “so low” due to not knowing when they could leave hospital.^38^

#### 3.5.3 How experiences of delay affect physical health

Due to a lack of movement and loss of independence, patients expressed concern about deterioration in their general health while in hospital^38^ and an increased risk of bed sores.^35^, ^36^, ^37^ Patients also reported that pressure to discharge them due to bed shortages meant that they had not recovered sufficiently prior to discharge^40^ and that their needs had not been addressed effectively.^39^ In some cases, this led to avoidable readmissions to hospital.^41^

#### 3.5.4 Experiences of staff

Discharge delays caused stress for staff for several reasons: they lengthened waiting lists (which the staff had responsibility to reduce) and created pressure for some patients to be discharged home, which in turn created frustration and guilt among staff who felt patients were being pressured to leave hospital.^40^ The strong management focus on reducing delayed discharges made staff feel “under the cosh”^35^, ^36^, ^37^ and adversely affected interprofessional relationships. The consultants and managers concerned with achieving government targets were most likely to pressure other staff to discharge patients and to become “disillusioned” about their care role^41^ because they were preoccupied with discharging patients, rather than providing care to those in need.^35^, ^36^, ^37^, ^41^, ^42^

Moreover, some procedures for addressing delayed discharge were perceived by staff to have “systematized” or dehumanized patients.^41^ Finally, some health professionals reported negative reactions towards patients, including “blame” for contributing to delays.^20^, ^21^, ^22^, ^34^, ^37^, ^38^ This exacerbated patients’ negative feelings about delays. Thus, staff responses to delay could aggravate patients’ negative experience of care caused by the length of the delay itself.

#### 3.5.5 Experiences at the hospital level

A number of organizational effects of delayed discharge were described. Adverse effects on the hospital culture included “poor mood on the ward,” which, in turn, had a knock‐on effect on the mood of patients’ receiving care.^43^

At the local system level, delays in transfer between health and social care providers contributed to “blame” and mistrust in interorganizational relationships.^35^, ^36^, ^37^ A hospital social worker described the “extra pressure” and “flak” they received from other staff in relation to delays.^35^, ^36^, ^37^ Information sharing between health and social care was also undermined by delays.^35^, ^36^, ^37^ Furthermore, use of “Section 5” in England—which gives notice of the proposed discharge date to social services and can trigger fines—had a negative effect on relations between health and social care, demotivating staff and causing tensions where this measure was used to pressure social workers to find placements.^35^, ^36^, ^37^

### 3.6 Summary of the health economic studies

The features of the 19 health economic studies are summarized in Table 4. Six studies refer to the UK and 5 to the United States; others are from the Netherlands, Ireland, France, Switzerland, Spain, Portugal, Greece and Australia. The 4 types of costs associated with delayed discharge were as follows: (i) costs due to patients occupying beds after they are medically fit for discharge, (ii) costs related to delays in admission to hospital that may occur where beds are still occupied by those delayed^35^, ^36^, ^37^, (iii) costs for nursing staff to produce reports and making phone calls to arrange discharge^41^, and (iv) administration costs associated with interventions designed to address delays.^35^, ^36^, ^37^

**Table 4:** Summary of the health economic studies

Author	Country	Type of service	Types of cost included	Differences between delay and non‐delay in terms of costs	Method of economic evaluation	Number of inappropriate days
Bartolome (2004)^45^	Spain	Centres of public primary, private clinics and A&E departments	Health service resources use	LOS of inappropriate admissions were 8.9+ −4.1 d compared to the planned 5.4+ −3.6 d. Reducing the hospital stay would decrease the cost of treating community‐acquired pneumonia by 8%. This would translate into a reduction in costs of 17.4% annually	Cost of illness analysis	In total 242 avoidable bed‐days
Basso (2009)^52^	The UK	Orthopaedic unit	Materials and drugs, staff costs, cost of bed occupancy	32 delayed patients: 19 lists required £38 703; 14 elderly patients (+7 younger): 11 lists required £22 407; 32 patients: 86 d: £17 200; 14 elderly (+7 younger): 51 d: £10 200	Cost analysis	86 d of bed occupancy
Brasel (2002)^56^	The USA	Trauma services	Hospital costs	The average cost per delay patient was $39 103 (range, $8983 to $103 861). The average cost per timely patient was $24 414 (range, $3930‐91 717).	Cost of discharge	
Buist (2014)^46^	Australia	Acute hospitals	Bed‐days	The cost for non‐medical bed‐days was estimated to be $764 800.	Cost analysis of health‐care utilization using the National Hospital Cost Data Collection results for NWAHS Hospitals from Round 14 (2009‐10)	475 (33%) of 1438 total bed‐days were for non‐medical reasons.
Macedo‐Vinas (2013)^55^	Switzerland	Primary and tertiary care	LOS, cost per bed, direct costs of MRSA infection	Cost estimation of additional CHF 30 866 per episode	Microcosting for the cost analysis; no economic evaluation	15.3 d
Carter (2002)^30^	The UK	Acute care	Delay days per annum, additional cost to the health service (expressed as £ and % of annual neurorehabilitation budget)	Delay days per annum 2844 for a total extra cost of £176 300 (23.5% of annual budget)		Not reported
Coughlan (2001)^49^	Ireland	Hospital	Costs for general hospital: elective operations waiting lists, emergency department overnight stays, cancellations of elective theatre, hospital funding; finances	Not stated	A cost analysis was not performed. They only assess the potential loss in terms of bed occupancy, surgery procedures, overnight stay in A&E	Not stated
Hwabejire (2013)^32^	The USA	Trauma unit	LOS costs (hospital cost)	The ExProH patients’ LOS was over 3 times longer and hospital cost 3 times higher (mean, $54 646 vs $18 444, respectively; *P* < .001)	Not stated	23 d vs 7 d
Johnson (2013)^53^	The USA	Surgical Intensive Care Unit (SICU)	Hospital costs	Over a population of 731 patients transferred from surgical intensive care, they estimated that transfer to the floor was delayed in 22% of cases (mainly due to lack of available beds in the ward), with delays from 1 to 6 d (mean, 1.5 d; median, 2 d) The cost associated with delays in transfer was estimated to be $581 790 for the entire study period, or $21 547 per week	Cost analysis	From 1 to 6 d
Kritikou (2016)^54^	Greece		Cost of length of stay, diagnostic tests, medications, hospital staff, and overhead services	Over 784 patients treated for stroke in a university hospital in Athens between 2003‐2009 they estimate the delayed discharge had a mean cost of €362 per patient	Bottom‐up cost analysis. No evaluation of outcomes or economic evaluation	
Landeiro (2016)^51^	Portugal	Orthopaedics	Daily cost of hospital stay; costs of a private care home, a private rehabilitation unit, domiciliary care services, formal and informal carer and required equipment. As the time horizon was 1 y, no discount rate was applied	The moderate risk group of patient registered additional costs per patient of €532 per extra day whereas the high risk/socially isolated group costs €905 per patient	Unit costs from national databases were used to estimate costs of delayed discharges	419 bed‐days lost. Patients with moderate risk of social isolation spent, on average, an additional 1.5 (95% CI: −0.5 to 3.3) days of delayed discharge compared to patients with a low risk of social isolation, ceteris paribus, while those with high risk/socially isolated spent an additional 2.6 (95% CI: 0.5 to 4.7) days on average
Menand (2015)^47^	France			For an older patient, the median cost of the hospital stay was € 3606.5 [€2498.1; €4994.2] for inappropriate admissions vs €4399.2 [€2862.8; €6 348.2] euros for appropriate admissions (*P* = .18)	Cost analysis from the hospital cost perspective	121 admissions
Mould‐Quevedo (2009)^48^	Mexico		Hospitalization costs (drugs, laboratory and radiologic examinations, intervisits to other specialists, procedures, emergency and administrative expenses). Third‐party payers	An appropriate hospitalization costs US $1497.2 (95% CI: US $323.2‐US $4931.4) per patient, while an inappropriate hospitalization resulted in US $2323.3 (95% CI: US $471.7‐US $6198.3), per patient	Direct medical costs associated with appropriate and inappropriate hospitalization estimated. Third payer perspective	5.1% (n = 198) of the 3891 d of hospital stays were classified as inappropriate
Niemeijer (2010)^58^	The Netherlands	Traumatology Department	LOS, admissions	118 additional admissions, at a cost of €176 400	Simple cost analysis is performed in the discussion	3.2 d
Hendy (2012)^44^	The UK	Medical and surgical / acute admissions unit		21% of inpatient stays were delayed discharge. The cost of an extra day was 565 sterling pounds per patient; 77% of delays due to provision of social and therapy requirements		
Polder (2003)^67^	The Netherlands		Societal perspective adopted to assess the costs, using a bottom‐up approach. Detailed measurement of investments in manpower, equipment materials, housing and overhead. Medical costs and private costs (informal care and travelling)	Average costs during the 4 mo after incidence of hip fracture were €14 281 for early discharged patients (€1057 less compared with conventionally discharged patients €15 338). Not statistically significant and with huge variation. A shift in costs from hospital to nursing home is caused by early discharge. Hospital costs were reduced by €2812 p < .001 nursing home costs increased by €1290 p < .001 on average	Costs examined from societal perspective using a bottom‐up methodology	
Thomas (2005)^57^	The USA	Traumatology	Cost of trauma patients admitted between 2001‐2003	For patients experiencing a delay in discharge from hospital, total hospital charges for excess bed‐days were $2 455 703 per year and total costs were $715 403 per year		1 in 25 patients admitted to the trauma centre, experienced an average of 6 d of delay in discharge, mainly attributable to challenges in patient placement (eg rehabilitation or subacute hospital bed not available)
Schwartz (2015)^68^	The USA		Cost for laparoscopic cholecystectomy;	The mean cost per person of laparoscopic cholecystectomy on hospital day 1 was $11 087. An incremental $2439 is paid 1 d after, $4146‐3 d after, $5735‐3 d after, etc	Cost analysis using hospital‐specific cost‐to‐charge ratios	34.5% of patients have a delayed surgery
Soria‐Aledo (2009)^50^	Spain	General	Cost of inappropriate stay	The cost of inappropriate admissions and stays in a sample of 725 hospitals admissions and 1350 hospital stays was €147 044. This represents €2 125 638 per year when extrapolated to the whole hospital		

Almost all studies provide evidence on the cost of patients occupying beds despite being medically fit for discharge. The average cost of an extra day varied according to ward and specialty: it is estimated at around £200‐£565 per patient per day. In a prospective study^44^ in 1 teaching hospital in London, 21% of the cohort's inpatient stays were discharged late, with an estimated average cost per patient of £565. This translates into a cost of more than £100 000 per year for a London ward of 30 beds.

At least 4 studies referred to the cost of inappropriate admissions for specific health conditions of surgical procedures.^45^, ^46^, ^47^, ^48^

An Irish study^49^ estimated an average of extra 8653 bed‐days per year by elderly patients that were waiting to be placed in long‐term care facilities. These extra days represent a loss in terms of opportunity cost, as they could be used for other interventions or to avoid overnight stays in A&E.^50^ Delayed discharge seems to be positively correlated with social isolation and referral to a public‐funded rehabilitation unit, whereas being admitted from an institution appears to be a protective factor for older patients presenting with hip fracture. According to a recent prospective study,^51^ conducted in Portugal on 278 patients admitted to a university hospital's orthopaedic ward, between 11.2% and 30.7% of total hospital costs could be saved by avoiding delays. In this study, unit costs from national databases were used to estimate the costs of delayed discharge: delayed discharge affected 22.3% of patients, causing a loss of 419 bed‐days (11.5% of total length of stay). They estimated that between 11.2% and 30.7% of total costs (€ 2352 and 9317 per delayed patient) were due to delayed discharge.

Delayed discharge caused cancellations of elective operations due to blocked beds, delaying operations and increasing costs of subsequent delays. When operating time is reduced or unavailable, this inevitably translates into delays in treatment and discharge for some patients.^52^ Delayed discharge can cause a bed‐block in surgical and medical wards causing delays in transfer within hospital.^53^, ^54^ Prolonged length of stays (LOS) is more likely to increase the risk of infections and therefore the costs associated with infections treatment.^55^

A US study^56^ estimated that one of the causes of delayed discharge was the lack of rehabilitation beds and that this would cause an additional hospital cost of $14 599 per delayed patient. In another prospective US study^57^ conducted in a trauma university hospital, all admitted patients between 2001 and 2003 were prospectively evaluated for the occurrence of delayed discharge: 1 in 25 patients had on average 6 days of delay in discharge, mainly attributable to challenges with patient placement, including absence of a rehabilitation or subacute hospital bed. In Massachusetts,^32^ patients with excessively prolonged hospitalization (ExProH) incurred higher hospitalization cost (mean, $54 646) compared with non‐ExProH patients (mean, $18 444). Therefore, to improve efficiency in a trauma system, it will be necessary to implement changes from acute care through to rehabilitation.

Discharge delays can have an impact not only on other admissions, but also on many other hospital services, including staff workload, physiotherapy, medical or surgery review, radiology, laboratory, pharmacy, transport, social and therapy services. In a prospective study conducted in London, they estimated that the repercussion of delayed discharge on other services can cost £0.5 million annually.^44^

In the Netherlands,^58^ an intervention to improve the discharge process reduced almost 50% of the inappropriate hospital stay, with a consequent improvement in trauma care quality and financial efficiency.

## 4 DISCUSSION

This is the first systematic review of the literature to take a comprehensive perspective on the impact of delayed discharge on patients, staff, and hospitals; and of their interrelationships.^3^ For patients, the main adverse outcomes are an association with an increased risk of mortality, hospital‐acquired infections, mental ill health and reductions in patients’ mobility and activities of daily living. For health‐care staff, the stress, diversion from a primary focus on patient care and deleterious interprofessional relationships, all have further harmful implications for patients’ health and well‐being. Finally, in addition to the impact on inpatient costs, we describe the economic repercussions for other services.

### 4.1 Strengths and limitations

This study's major strength is the collation of results from different primary studies to highlight the multiple and interrelated effects of delayed discharge on patients, health professionals and hospitals. Previous research focussed on specific aspects of delay. Thus, few qualitative studies fully captured experiences of delay from patient, staff and hospital perspectives,^35^, ^36^, ^37^ while quantitative studies focussed on a limited number of health outcomes. By examining the impacts of delayed discharge within and across the health system, this review revealed interactions and sequelae. For example, the physical or emotional impacts on patients can lead to disengagement from care or discharge planning processes which could contribute to further adverse impact on patients’ health. Furthermore, patients’ negative experiences were sometimes exacerbated by staff reactions to prolonged hospital stay, when the quality of the care they provided could deteriorate due to the stress of dealing with delays or because they “blamed” patients for delays. Thus, this review adds to previous literature by highlighting the “knock‐on” effects of delay within the system.

Our findings on the impact of delayed discharge on hospital readmissions are in line with existing studies, including evidence that delays result in other patients being discharged prematurely^43^ and that some health and social needs were neglected.^39^ However, previous studies underestimated the economic impact of delayed discharge by assessing individually specific type of costs, such as the extra LOS, the cost of acquired infections or the cost of cancelled interventions. In this review, we highlight the importance of summing up all those costs and of including a more comprehensive list that takes into account the organizational costs, the repercussions on other services and the potential societal costs.

A major weakness of our findings is the lack of comprehensive evidence on delayed discharge from a single country's health system. This might have more easily allowed exploration of structural or policy related explanations. In the absence of sufficient data for intranational examination, we followed the well established route of examining research from OECD countries.^59^ The OECD has been a prime source of international comparative data on health systems for many decades. A second potential weakness is the low methodological quality: we judged that two thirds of the quantitative and qualitative studies had low to moderate quality; this potentially limits the reliability of the results. Thirdly, some studies which assessed the impact of prolonged LOS without reporting the reasons for delay might have been excluded. Fourthly, most studies did not identify the type of infection acquired and how associated costs were measured, making it challenging to assess the impact of delays accurately. Finally, we limited our review to papers written in English which might have excluded relevant findings, although this represented <5% of references identified for full‐text assessment.

### 4.2 Implications for practice, policy and research

Recent policies on delayed discharge advocate “system‐level” approaches to addressing delays, for example encouraging shared leadership and integration across health and social care.^6^, ^60^, ^61^ Previous research has found examples of joint working between health and social care that improved working relationships and facilitated ownership of delays.^35^, ^36^, ^37^ This review confirms the importance of system‐level approaches that address the effects of delay at multiple levels. It is recognized that delayed discharge is a contested concept, due to differing interpretations in policy and among health and social care providers on the reasons for, and measurement of, delays.^62^ For the purposes of this review which included studies from different countries, we have defined delayed discharge as a stay for a patient who is beyond being deemed “medically fit” to leave hospital, but is unable to do so for non‐medical reasons. However, we acknowledge that the nature of delayed discharge is not fixed, but varies across health systems and is locally negotiated by health and social care providers in response to different policy and organizational environments.^63^ For example, NHS England's definition of a patient that is ready for discharge includes the safety and appropriateness of the discharge destination as well as clinical considerations, whereby a discharge may only occur when (i) a clinical decision has been made that the patient is ready for transfer, (ii) a multidisciplinary team decision has been made that the patient is ready for transfer, and (iii) the patient is safe to discharge/transfer.^64^ The variety of reasons for delay, including those linked to local and national policy, may add to the self‐perceptions of patients, particularly frail older people, of being “bed blockers” that contribute to delays, which may adversely affect their health by causing further stress and anxiety linked to feeling at fault for delays.^35^, ^36^, ^37^, ^43^ Even where there is variation in defining delays in discharge there is broad acceptance among professionals working in the health service that it continues to be a significant problem that impacts the provision of care.^65^ Our findings provide renewed emphasis for the need to standardize the approach to measuring delays and invest in delayed discharge the issue as a priority given its impact not only on patients’ health and experiences of care, but also on staff well‐being, interprofessional relationships and information sharing; and on distal (in addition to proximal) costs.^66^

We have highlighted that the real cost of delayed discharge must include unit level (eg LOS or infection costs or cancelled operations), organizational and local system‐level impacts. However we should also consider other costs that have not been quantified yet (eg the impact on staff morale, staff turnover, agency fees, cost of social care, nursing homes), but could have a huge economic impact. Delayed discharge represents an opportunity cost that is not necessarily equal to the forgone margin from a new admission. There are many repercussions on other services, such as staff, physiotherapy, radiology, pharmacy, surgery, occupational therapy, laboratory and lack of downstream beds that should be considered when assessing the costs of delayed discharge.^44^ Further attention should also be placed on societal costs related to productivity losses due to delay discharge of patients in working age or their caregivers, transport costs to visit delayed patients and impacts on other sectors.^67^

Finally, to assess the longer term impact of delays, prospective cohort studies are required that combine routine data from health and social care databases and supplement this with additional process and outcome data.

## DISCLAIMER

The views expressed are those of the author(s) and not necessarily those of the NHS, the NIHR or the Department of Health.

## AUTHORS' CONTRIBUTIONS

All authors have made substantial contributions to this study. ARG, ST, JT and RR were responsible for the conception and design of the study. ARG and JT were responsible for the design of the search strategies. ARG, ST, EP and EH screened titles and abstracts of retrieved references and full‐text documents. ARG, ST, EP and EH were responsible for the analysis and interpretation of data. All were responsible for drafting the article or revising it critically for important intellectual content.

## CONFLICT OF INTEREST

None declared.
