# Supplementary material for: Impact and experiences of delayed discharge: A mixed‐studies systematic review
Source: Health Expect. 2017 Sep 12;21(1):41–56. doi: 10.1111/hex.12619 (PMC5750749; doi:10.1111/hex.12619)
Supplement: Supplementary file 1 [file HEX-21-41-s001.docx]

## Appendix

### Search strategies

### Methodological quality of the quantitative papers and tool used to assess qualitative studies

| **Quality assessment of quantitative studies** | | |  |  |  |  |  |
| --- | --- | --- | --- | --- | --- | --- | --- |
| **Authors (year)** | **1. Was an appropriate comparison group used? YES/NO** | **2. Were confounders considered in analysis? YES/NO** | **3. Was this a representative sample of the stated population of interest? YES/NO** | **4. Were reliable measures of Delayed discharge/inappropriate stay used? YES/NO** | **5. Were reliable measures of ADL, Comorbidity, etc. used? YES/NO** | **6. Do you have concerns about the relevance of this study to the review in a health service context? YES/NO** | **Global Score (Risk of bias)** |
| Carter (2002) | NO | NO | YES | YES - staff's criteria | YES | YES | High |
| Challis (2014) | YES - Timely discharge | YES | YES | YES - ‘SitReps’ reporting system | YES | YES | Low |
| Costa (2012) | NO | YES | YES | YES - staff's criteria | YES | YES | Moderate |
| Ingold (2000) | YES - Appropriate stay | YES | YES | YES - AEP | YES | NO | Moderate |
| Jasinarachchi (2009) | YES - Appropriate stay | YES | YES | YES - As DH recommends | YES | YES | Moderate |
| Moeller (2006) | YES - Non-delay | YES | YES | YES - Checklist | YES | YES | Low |
| Rosman (2015) | NO | YES | YES | YES - staff's criteria | YES | NO | Moderate |
| Umarji (2006) | NO | NO | YES | YES - staff's criteria | YES | YES | High |
| Young (2010) | YES | YES | YES | Unclear | YES | YES | Low |
| Hwabejire (2013) | YES | NO | YES | YES - staff's criteria | YES | NO | High |

Criteria used for assessing quality of the qualitative studies

| 1. Were steps taken to increase rigour in the sampling? | Yes, a fairly thorough attempt was made    Yes, several steps were taken    Yes, a few steps were taken    No, not at all/ Not stated/Can’t tell |
| --- | --- |
| 2. Were steps taken to increase rigour in the data collected? | Yes, a fairly thorough attempt was made    Yes, several steps were taken    Yes, minimal few steps were taken    No, not at all/ Not stated/Can’t tell |
| 3. Were steps taken to increase rigour in the analysis of the data? | Yes, a fairly thorough attempt was made    Yes, several steps were taken    Yes, minimal few steps were taken    No, not at all/ Not stated/Can’t tell |
| 4. Were the findings of the study grounded in/ supported by the data? | Good grounding/support    Fair grounding/support    Limited grounding/support |
| 5. Please rate the findings of the study in terms of their breadth and depth. | Limited breadth or depth    Good/fair breadth but very little depth    Good /fair depth but very little breadth    Good/fair breadth and depth |
| 6. To what extent does the study privilege the perspectives and experiences of health care professionals and patients/carers that are relevant to the NHS and comparable health systems? | Not at all    A little    Somewhat    A lot |
| 7. Overall, what weight would you assign to this study in terms of the reliability/trustworthiness of its findings? *Guidance: Use answers given to questions 1 to 4.* | Low    Medium    High |
| 8. What weight would you assign to this study in terms of the usefulness of its findings for this review? | Low    Medium    High |

### Data extraction form


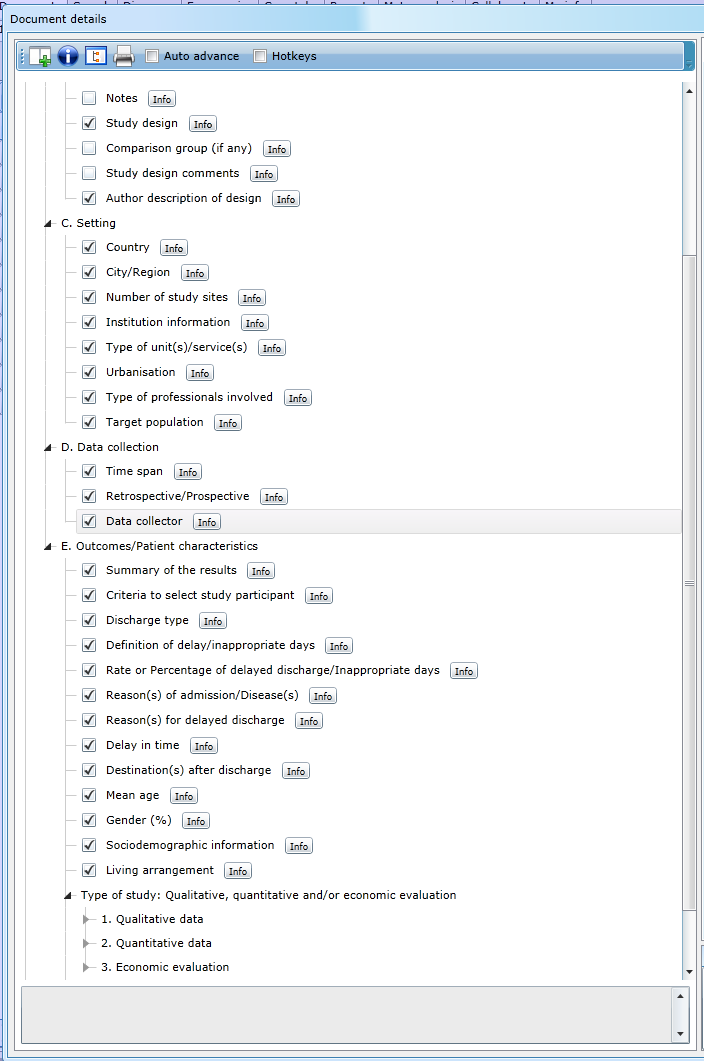


| **Section/topic** | | **#** | | **Checklist item** | **Reported on page #** |
| --- | --- | --- | --- | --- | --- |
| **TITLE** | | | | |  |
| Title | | 1 | | Identify the report as a systematic review, meta-analysis, or both. | 1 |
| **ABSTRACT** | | | | |  |
| Structured summary | | 2 | | Provide a structured summary including, as applicable: background; objectives; data sources; study eligibility criteria, participants, and interventions; study appraisal and synthesis methods; results; limitations; conclusions and implications of key findings; systematic review registration number. | 2 |
| **INTRODUCTION** | | | | |  |
| Rationale | | 3 | | Describe the rationale for the review in the context of what is already known. | 3 |
| Objectives | | 4 | | Provide an explicit statement of questions being addressed with reference to participants, interventions, comparisons, outcomes, and study design (PICOS). | 3 |
| **METHODS** | | | | |  |
| Protocol and registration | 5 | | Indicate if a review protocol exists, if and where it can be accessed (e.g., Web address), and, if available, provide registration information including registration number. | | Prospero  CRD42016035256 |
| Eligibility criteria | 6 | | Specify study characteristics (e.g., PICOS, length of follow-up) and report characteristics (e.g., years considered, language, publication status) used as criteria for eligibility, giving rationale. | | 4 |
| Information sources | 7 | | Describe all information sources (e.g., databases with dates of coverage, contact with study authors to identify additional studies) in the search and date last searched. | | 4 |
| Search | 8 | | Present full electronic search strategy for at least one database, including any limits used, such that it could be repeated. | | (Appendix 1) |
| Study selection | 9 | | State the process for selecting studies (i.e., screening, eligibility, included in systematic review, and, if applicable, included in the meta-analysis). | | 4 |
| Data collection process | 10 | | Describe method of data extraction from reports (e.g., piloted forms, independently, in duplicate) and any processes for obtaining and confirming data from investigators. | | 4 |
| Data items | 11 | | List and define all variables for which data were sought (e.g., PICOS, funding sources) and any assumptions and simplifications made. | | 4-5 |
| Risk of bias in individual studies | 12 | | Describe methods used for assessing risk of bias of individual studies (including specification of whether this was done at the study or outcome level), and how this information is to be used in any data synthesis. | | 4 |
| Summary measures | 13 | | State the principal summary measures (e.g., risk ratio, difference in means). | | - |
| Synthesis of results | 14 | | Describe the methods of handling data and combining results of studies, if done, including measures of consistency (e.g., I^2^) for each meta-analysis. | | 4-5 |

Page 1 of 2

| **Section/topic** | **#** | **Checklist item** | **Reported on page #** |
| --- | --- | --- | --- |
| Risk of bias across studies | 15 | Specify any assessment of risk of bias that may affect the cumulative evidence (e.g., publication bias, selective reporting within studies). | - |
| Additional analyses | 16 | Describe methods of additional analyses (e.g., sensitivity or subgroup analyses, meta-regression), if done, indicating which were pre-specified. | - |
| **RESULTS** | | |  |
| Study selection | 17 | Give numbers of studies screened, assessed for eligibility, and included in the review, with reasons for exclusions at each stage, ideally with a flow diagram. | 5 |
| Study characteristics | 18 | For each study, present characteristics for which data were extracted (e.g., study size, PICOS, follow-up period) and provide the citations. | 6-7 |
| Risk of bias within studies | 19 | Present data on risk of bias of each study and, if available, any outcome level assessment (see item 12). | 7 and (Appendix 3) |
| Results of individual studies | 20 | For all outcomes considered (benefits or harms), present, for each study: (a) simple summary data for each intervention group (b) effect estimates and confidence intervals, ideally with a forest plot. | 8-9, 12-13, 17-19 |
| Synthesis of results | 21 | Present results of each meta-analysis done, including confidence intervals and measures of consistency. | - |
| Risk of bias across studies | 22 | Present results of any assessment of risk of bias across studies (see Item 15). | - |
| Additional analysis | 23 | Give results of additional analyses, if done (e.g., sensitivity or subgroup analyses, meta-regression [see Item 16]). | - |
| **DISCUSSION** | | |  |
| Summary of evidence | 24 | Summarize the main findings including the strength of evidence for each main outcome; consider their relevance to key groups (e.g., healthcare providers, users, and policy makers). | 20 |
| Limitations | 25 | Discuss limitations at study and outcome level (e.g., risk of bias), and at review-level (e.g., incomplete retrieval of identified research, reporting bias). | 20 |
| Conclusions | 26 | Provide a general interpretation of the results in the context of other evidence, and implications for future research. | 21 |
| **FUNDING** | | |  |
| Funding | 27 | Describe sources of funding for the systematic review and other support (e.g., supply of data); role of funders for the systematic review. | - |

*From:*  Moher D, Liberati A, Tetzlaff J, Altman DG, The PRISMA Group (2009). Preferred Reporting Items for Systematic Reviews and Meta-Analyses: The PRISMA Statement. PLoS Med 6(7): e1000097. doi:10.1371/journal.pmed1000097

For more information, visit: **www.prisma-statement.org**.

Page 2 of 2
